# A Cross-Sectional Study Evaluating the Quality of AI-Generated Patient Education Guides on Diet and Exercise for Diabetes, Hypertension, and Obesity Using ChatGPT-4o, Google Gemini 1.5, Claude Sonnet 4, Perplexity, and Grok

**DOI:** 10.7759/cureus.106221

**Published:** 2026-03-31

**Authors:** Charane Sree Aenuginti, Tejaswi Mangalagiri, Naga Sai Gouthami Gurujala, Vasantha Kumar Polisetty, Prashasti Sharma, Yashaswi Guntupalli, Subhash K Reddy

**Affiliations:** 1 Internal Medicine, Sri Padmavathi Medical College for Women, Tirupati, IND; 2 Internal Medicine, Meenakshi Medical College Hospital and Research Institute, Kanchipuram, IND; 3 Internal Medicine, NRI Medical College, Chinakakani, IND; 4 Oncology, Peterborough City Hospital, Peterborough, GBR; 5 Internal Medicine, Sri Venkateswara Institute of Medical Sciences (SVIMS), Tirupati, IND; 6 Cardiology, Lutheran Hospital, Fort Wayne, USA

**Keywords:** artificial intelligence (ai), cross-sectional studies, diabetes, diet, excercise, hypertension, large language model (llm), obesity, patient education guide, quality evaluation

## Abstract

Background: Diabetes, obesity, and hypertension are common chronic conditions in which the role of lifestyle alteration is central to control. Education materials may accompany these interventions, but will only be helpful if clear and credible. Since the development of large language models (LLMs) like ChatGPT and Google Gemini, the potential of contributing to the production of health education materials should be seriously taken into account.

Aim: The aim of this study is to conduct a cross-sectional comparison of five LLMs (ChatGPT-4o, Google Gemini 2.5, Claude Sonnet 4, Grok 3, and Perplexity) in generating patient education brochures on diet and exercise for diabetes, hypertension, and obesity, evaluating their readability, originality, and reliability.

Primary objective: The primary objective is to compare the readability and reliability of AI-generated patient education materials.

Secondary objective: The secondary objective is to assess lexical complexity and originality of generated content.

Methods: This cross-sectional study used standardized questions to generate brochures based on each response provided by the LLMs. Outputs were evaluated for readability (Flesch-Kincaid test), word complexity, novelty (PapersOwl plagiarism software), and consistency (modified DISCERN instrument). Descriptive statistics and one-way ANOVA were used, where p < 0.05 was deemed significant.

Results: ChatGPT produced the shortest and most readable content, evidenced by its lowest grade level (5.2 ± 0.8) and highest Flesch Reading Ease rating (70.0 ± 5.1). Gemini and Claude produced longer, more elaborate brochures that received higher reliability ratings (3.0 ± 0.0 and 3.0 ± 1.0, respectively) but were at higher reading levels (≈ 9th grade). Grok obtained medium for all the measures, while Perplexity produced shorter responses (≈ 444 words) but the lowest reliability score (1.3 ± 0.6). There were no major differences in originality scores across the tools.

Conclusion: Every model had a strength: ChatGPT in readability, Gemini and Claude in reliability, Grok in balance, and Perplexity in conciseness. Every model demonstrated at least one parameter where it outperformed the others. The results validate LLMs in terms of producing patient-comprehensible leaflets, but human editing, updation with the latest guidelines, and human supervision will be needed before clinical application.

## Introduction

Diabetes, hypertension, and obesity are the world's three most prevalent chronic diseases, all of which result in disability and premature death. Over 530 million people suffer from diabetes [1], over one billion from hypertension [2], and nearly 650 million from obesity [3]. These are not numbers but a daily concern for tens of millions of households across the globe. Changes in lifestyle like diet and exercise are still the mainstay of management [4]. But success is regularly contingent upon adherence to and utilization of advice provided by patients [5].

Patient education materials are created to bridge this gap [6]. Most brochures and Internet resources are, nonetheless, written at more than average reading level [7]. Although some patients are at the sixth- to eighth-grade level, most information presented is in ninth grade and above. Low readability and poor reliability discourage patient interest, compliance, and shared decision-making [8,9].

Artificial intelligence (AI) has of late been viewed as a possible means of filling such gaps [10,11]. Large language models (LLMs) like ChatGPT (OpenAI) and Gemini (Google DeepMind) can produce plain text descriptions, transfer tone for varying literacy levels, and lower the cost and time of creating content. Initial research indicates such models can enhance readability, but accuracy, citation reliability, and the ability to convey empathy are issues.

This study is a comparison of five widely used LLMs like ChatGPT-4o (OpenAI, San Francisco, USA) [12], Google Gemini 2.5 (OpenAI, San Francisco, USA) [13], Claude Sonnet 4 (Anthropic, San Francisco, USA) [14], Grok 3 (xAI, USA) [15], and Perplexity (Perplexity AI, San Francisco, USA) [16] in the production of patient education leaflets on diet and exercise in diabetes, hypertension, and obesity. Their quality was assessed for readability, lexical complexity, novelty, and validity to gauge practical use in patient education. 

Aim

This study aims to conduct a cross-sectional comparison of five LLMs (ChatGPT-4o, Google Gemini 2.5, Claude Sonnet 4, Grok 3, and Perplexity) in generating patient education brochures on diet and exercise for diabetes, hypertension, and obesity.

Primary objective

The primary objective is to compare readability and reliability across models.

Secondary objective

The secondary objective is to evaluate lexical complexity and originality and assess applicability in patient education.

## Materials and methods

Study design

This was a cross-sectional study conducted in July 2025. As no human participants were involved, ethics approval was not required.

Data collection

Three common non-communicable diseases, diabetes, hypertension, and obesity, were selected because lifestyle interventions are central to their management. Five widely accessible LLMs were chosen for evaluation: ChatGPT-4o, Google Gemini 2.5, Claude Sonnet 4, Grok 3, and Perplexity AI.

For each condition, the following standardized prompt was given: “Write a patient education guide on diet and exercise for [disease name].” This process was repeated separately for diabetes, hypertension, and obesity, generating a total of 15 brochures. All outputs were exported in Microsoft Word format for analysis.

Model generation settings

All models were accessed through their publicly available web interfaces using default settings. No manual adjustments were made to parameters such as temperature, token limits, or randomness. Each prompt was executed once per model for each condition without regeneration.

Data preprocessing

Generated outputs were exported into Microsoft Word format without modification. Formatting elements such as headings, bullet points, and paragraph structures were retained. No cleaning or editing was performed prior to readability or originality analysis.

DISCERN scoring procedure

Reliability was assessed using a modified DISCERN instrument. All evaluations were conducted independently by a single author. No blinding or inter-rater validation was performed.

Evaluation metrics

Evaluation metrics used to assess LLM-generated brochures are described in Table 1. Each brochure was assessed using the following parameters.

**Table 1:** Evaluation metrics used to assess LLM-generated brochures LLM: Large language model

Metric	Tool/Scale Used	Purpose
Readability	FK test [17]	Word/sentence counts, grade level, ease
Lexical complexity	Average syllables/word	Approximate technicality of vocabulary
Originality	PapersOwl similarity check [18]	Detect overlapping/plagiarized content
Reliability	Modified DISCERN tool [19]	Evaluate structure, risks, benefits, advice

Each brochure was evaluated using predefined quality parameters. Readability and text complexity were assessed using the Flesch-Kincaid readability calculator (FK test) [17], which reports word count, sentence count, average words per sentence, average syllables per word, grade level, and reading ease score. Lexical complexity was approximated using average syllables per word and grade level. Originality was assessed using the PapersOwl plagiarism checker to detect overlapping content [18]. Reliability was evaluated using the modified DISCERN tool [19], a validated five-item checklist for written health information, in which each “yes” response scored one point for a maximum possible score of five.

The strengths and weaknesses of each brochure were determined based on their performance across the evaluation parameters described. Brochures with higher readability scores (lower grade level and higher ease score), lower similarity percentages, and higher reliability scores were considered stronger educational resources. Those with lower readability or reliability, higher lexical complexity, or substantial overlap were considered weaker. All assessments were independently conducted by one of the study authors.

Statistical analysis

This cross-sectional study used standardized questions to create brochures from every response alternative. Outputs were evaluated for readability (FK test), word complexity, novelty (PapersOwl plagiarism software), and consistency (modified DISCERN instrument). Data from all outputs were compiled in Microsoft Excel (Version: 2508 Build 16.0.19127.20192, Microsoft Corporation, Redmond, United States) and analyzed using Python programming language (Version 3.12.3, Python Software Foundation, Wilmington, United States) [20,21]. Descriptive statistics and one-way analysis of variance (ANOVA) [22] were used, where a probability value (p-value) < 0.05 was deemed significant.

## Results

A total of 15 patient education brochures were generated by five LLMs for three conditions: diabetes, hypertension, and obesity. These were evaluated for readability, lexical complexity, originality, and reliability. The integrated results are presented in Table 2.

**Table 2:** Comparative readability, lexical complexity, similarity (originality), and reliability (modified DISCERN) of brochures produced by ChatGPT-4o, Google Gemini 2.5, Claude Sonnet 4, Perplexity, and Grok. *p-values <0.05 are considered statistically significant.

Parameter	ChatGPT4o	Google Gemini 2.5	Claude Sonnet 4	Perplexity	Grok	F-statistic	p-value
Words	450.67 ± 20.03	1570.67 ± 65.96	2460.67 ± 1865.38	443.67 ± 50.54	783.67 ± 154.10	3.22	0.0607
Sentences	64.67 ± 4.51	138.33 ± 4.51	245.33 ± 196.92	51.67 ± 10.21	96.33 ± 22.59	2.31	0.1285
Avg words per sentence	6.97 ± 0.32	11.37 ± 0.21	10.33 ± 0.55	8.73 ± 0.95	8.20 ± 0.30	31.79	0.0000*
Avg syllables per Word	1.53 ± 0.06	1.73 ± 0.06	1.77 ± 0.12	1.70 ± 0.10	1.80 ± 0.10	4.04	0.0333*
Grade level	5.23 ± 0.76	9.30 ± 0.78	9.27 ± 1.34	7.87 ± 0.93	8.83 ± 1.05	8.79	0.0026*
Ease score	70.03 ± 5.06	48.63 ± 5.05	46.90 ± 9.80	54.13 ± 7.78	46.23 ± 8.15	5.38	0.0142*
Similarity %	24.33 ± 21.35	23.23 ± 6.31	30.13 ± 15.39	27.27 ± 2.90	14.50 ± 8.41	0.64	0.6439
Reliability score	2.33 ± 0.58	3.00 ± 0.00	3.00 ± 1.00	1.33 ± 0.58	3.00 ± 0.00	4.80	0.0202*

A t-test was not performed for the data presented in Table 2. Since we compared five LLMs (ChatGPT-4o, Google Gemini 2.5, Claude Sonnet 4, Perplexity, and Grok) across multiple parameters (e.g., readability, lexical complexity, originality, and reliability), we used ANOVA rather than a t-test. A one-way ANOVA is the appropriate statistical test when comparing the means of more than two independent groups. The F-statistics and p-values reported in Table 2 represent the results of this ANOVA analysis. Statistical significance was set at p < 0.05. Important strengths and weaknesses of the five LLMs used in this study when generating patient education guides are listed in Table 3. 

**Table 3:** Strengths and weaknesses of the five LLMs in generating patient brochures LLMs: Large language models

Model	Strengths	Weaknesses
ChatGPT-4o	Simplest text, lowest grade level, highest ease score	Lower reliability score, less structured content
Gemini 2.5	Most reliable, well-structured brochures	Harder to read, higher literacy demands
Claude 4	Detailed, reliable, comprehensive	Complex wording, requires advanced reading
Grok 3	Balanced across readability and reliability	Moderate scores, not leading in any category
Perplexity	Short, concise brochures	Lowest reliability, limited coverage

The strengths and weaknesses of each brochure were determined based on their performance across the evaluation parameters described. Brochures with higher readability scores (lower grade level and higher ease score), lower similarity percentages, and higher reliability scores were considered stronger educational resources. Those with lower readability or reliability, higher lexical complexity, or substantial overlap were considered weaker. All assessments were independently conducted by one of the study authors.

Regarding readability and text complexity, sentence length varied significantly between models. ChatGPT used shorter sentences, averaging approximately seven words per sentence, whereas Gemini and Claude averaged more than ten words per sentence. ChatGPT’s brochures corresponded to the lowest grade level at 5.2 ± 0.8 and achieved the highest Flesch Reading Ease score at 70.0 ± 5.1 [23,24]. In contrast, Gemini and Claude required nearly a ninth-grade reading level and demonstrated ease scores below 50. Grok showed intermediate readability performance, while Perplexity generated shorter brochures with moderately complex vocabulary.

With respect to lexical complexity, vocabulary density differed across models. ChatGPT used simpler words, averaging 1.53 syllables per word, whereas Claude and Grok demonstrated higher lexical complexity, approaching approximately 1.8 syllables per word.

In terms of originality, no statistically significant differences were observed in similarity percentages across models. All tools maintained acceptable originality levels, ranging from 14 percent for Grok to 30 percent for Claude.

Regarding reliability, DISCERN-based scores were highest for Gemini, Claude, and Grok, each averaging 3.0 out of 5. ChatGPT scored 2.3 out of 5, while Perplexity demonstrated the lowest reliability score at 1.3 out of 5.

When results were stratified by disease category, performance patterns remained consistent across diabetes, hypertension, and obesity. ChatGPT consistently produced the most readable brochures, whereas Gemini and Claude maintained higher reliability scores. Perplexity underperformed across conditions, while Grok demonstrated balanced but moderate performance.

Overall, the findings highlight consistent trade-offs between readability and reliability across models. ChatGPT generated brochures that were easiest to read but less structured, whereas Gemini and Claude produced more reliable yet more complex materials. Grok offered balanced performance, and Perplexity produced concise but less dependable outputs. No statistically significant differences were observed in similarity percentages, and word and sentence counts did not significantly differ across models, indicating that content length was not a major differentiating factor.

## Discussion

This research suggests a consistent and clear trade-off in employing LLMs to generate patient education resources. Those models that produced shorter sentences and less complex vocabulary, like ChatGPT, read most easily with a fifth-grade average reading level and ease score of approximately 70. Gemini and Claude, however, produced longer, more sophisticated brochures that were more credible, performing well on DISCERN at an average value of 3 out of 5, but at the cost of almost a ninth-grade reading level. Grok exhibited moderate but balanced performance among subject topics, while Perplexity generated concise texts with simple scanning but the lowest reliability score of approximately 1.3. Such findings reinforce that no model outperforms all measures at all times.

Overall, the suggestion is that the usefulness of any model will be a function of the purpose to which patient education is to be put. If simplicity of use by low-literacy users is of greatest concern, ChatGPT's tone is beneficial. If thoroughness and reliability to form are of primary concern, Gemini or Claude may be better, but at the price of higher literacy expectations. Grok can serve as an accommodation option, but Perplexity's brevity is balanced only by shallow depth.

Besides model comparison, this research implies deeper issues with safely applying LLMs in health communication. The biggest challenge may be the potential for counterfeit or deceptive references [25-28]. While the text itself is transparent and convincing, false citations undermine credibility and might mislead practitioners and patients. AI-driven reference-checking tools and editorial safeguards will therefore be essential prior to general application. Another point of consideration is that readability scores, while helpful for large-scale comparisons, do not actually quantify understanding.

Low-grade-level scoring does not necessarily imply understanding, cultural relevance, or correct behavioral alteration. Field validation with patients is still the gold standard. In order to have safety guaranteed, a tiered process is advised: draft with guided prompting, use automated scans for citation and readability and then final clinician review and patient testing prior to release. This human-in-the-loop approach maintains efficiency through the addition of quality control. Ultimately, ethical and operational issues need to be dealt with [29]. The forceful voice of responses generated by AI can drive even minor mismatches into egregious errors, and patients can take draft content to be definitive medical recommendations. Open declarations of AI utilization, disclaimers, and clinical oversight are necessary protections. Originality and "AIgiarism" problems also prefer open recognition of AI participation in manuscript preparation. Despite these findings, the study should be interpreted with caution due to methodological constraints. The limited number of prompts and outputs may not capture the full variability of LLM responses. Additionally, the absence of multiple evaluators for DISCERN scoring introduces potential subjectivity. The lack of real-world validation further limits generalizability to clinical practice.

 Limitations

The study is a snapshot of how well LLMs performed over a one-week period in July 2025. Due to the high frequency of model updates, subsequent iterations might yield different results. Secondly, only readability, lexical complexity, originality, and reliability were tested. Other crucial dimensions such as empathy, cultural sensitivity, and emotional tone were not included [30]. Third, the research was not subject to end-user testing with patients or clinicians; although readability tests are helpful, they do not replace actual-world comprehension and usability testing. Last, adding bibliographic verification and clinician review, as proposed, could add time and resource requirements that may restrict practice scale-up. 

Additionally, the use of a single standardized prompt per condition may not fully capture variability in LLM-generated outputs. The study did not control for model generation parameters such as temperature or randomness, which may influence responses. All evaluations were conducted by a single reviewer, introducing potential subjective bias and limiting inter-rater reliability. Furthermore, interface conditions such as web-based access versus API use or free versus paid versions were not standardized and may affect reproducibility. The relatively small sample size may also limit statistical power, and findings should therefore be interpreted as exploratory.

## Conclusions

LLMs can generate patient education brochures that may be easier to read than some traditional materials; however, they are not yet fully reliable as stand-alone solutions. ChatGPT produced the simplest and most comprehensible materials during these tests, with Gemini and Claude offering more reliability but at the expense of more difficulty in reading. Grok was somewhere in between those features, and Perplexity produced shorter brochures but lacked structural depth. These trends were replicated in diabetes, hypertension, and obesity.

Before they can be adopted for use in the healthcare environment, LLM-authored guides must be submitted to a tiered quality-control process involving automated reference checking and readability evaluation, clinician review, and patient testing. When used within such safeguards, LLMs may have potential as drafting tools to support the development and maintenance of patient-centered health information. Continued development should extend testing to measures such as empathy, cultural sensitivity, and real-world grounding, to make certain that these tools augment but not replace human-led patient education.
